# Salinity-induced modulation of growth and antioxidant activity in the callus cultures of miswak (*Salvadora persica*)

**DOI:** 10.1007/s13205-012-0064-6

**Published:** 2012-04-24

**Authors:** Varsha Sharma, Kishan Gopal Ramawat

**Affiliations:** Department of Botany, Laboratory of Bio-Molecular Technology, M. L. Sukhadia University, Udaipur, 313001 India

**Keywords:** Antioxidant, Catalase, Salinity, *Salvadora persica*, Phenolics, Proline

## Abstract

Miswak (*Salvadora persica* Linn.) is a medium-sized tree, desert facultative halophytic plant. Besides edible fruits and non-edible seed oil, the plant contains several bioactive compounds like alkaloids, tannins, saponins and sterols related to food and cosmetic industries. In the present study, physiological responses and antioxidant potential under salinity stress were investigated in callus cultures of *S. persica* to evaluate its use as a source of antioxidant. The callus cultures were grown on MS medium supplemented with 0.5 mg/l each of 2,4,5-T and BAP, which could be established successfully by regular subcultures of slow growing callus on this medium for several months. Increased dry weight, soluble proteins, proline, soluble carbohydrates and CAT activity were recorded under NaCl stress in comparison to control cultures. The DPPH and FRAP antioxidant activities were gradually elevated in NaCl-treated callus, whereas SOD quenching was recorded maximum at 200 mM. A significant correlation between antioxidant capacity and phenol content was observed, indicating that phenolic compounds are the major contributors to the antioxidant potential in *S. persica*. These findings suggest that increased salinity stress caused elevated antioxidant potentials and the plants grown in such conditions may serve as potential source of antioxidant.

## Introduction

High salinity, drought, extreme light and temperature are the most serious environmental stresses impending plant growth and limiting crop productivity worldwide (Munns 2005). In salt affected plants, the result is primarily an ionic imbalance and hyper osmotic stress (Tester and Davenport 2003). The effect of this imbalance or disruption in homeostasis occurs at the cell as well as the whole plant levels. Finally, in extreme saline condition, molecular damage and growth arrest lead to death of plant (Jitesh et al. 2006). Salt-tolerant plants have evolved a variety of physiological responses that confer capacity for osmotic adjustment. These plants accumulate osmolytes such as glycine, betaine and proline that maintain the osmotic balance disrupted by the presence of ions in the vacuole (Wang et al. 2004). Investigations of halophytes indicated that significantly increased levels of proline and soluble carbohydrates are probably related to osmotic adjustment and the protection of membrane stability under salinity stresses (Megdiche et al. 2007). Proteins that accumulate in plants under saline conditions may provide a storage form of nitrogen that is re-utilized later and also play a role in osmotic adjustment.

In accordance with the established mechanism of ROS generation, oxidative stress has been reported in several plant species after NaCl stress treatment. Further, plant materials containing phenolic compounds are increasingly of interest for the food industry as they participate in the defense against ROS. Thus, drought-stressed plants might represent potential sources of polyphenols for economical use (Bettaieb et al. 2011). The correlation between antioxidant capacity and salt tolerance was demonstrated in a large number of plants, including salt-tolerant glycophytes and true halophytes such as *Beta maritime, Cassia angustifolia* and *Crithmum maritimum* (Li 2008).

*Salvadora persica* Linn. (Family Salvadoraceae) is a typical facultative halophytic plant, grows in arid regions from western India to Middle East as well as high saline lands along the sea coasts. It is an important plant of arid horticulture (edible fruits and non-edible seed oil) and forestry, hence micropropagation method has been developed (Phulwaria et al. 2011). The WHO recommends and encourages the use of chewing sticks of miswak as an effective oral hygiene procedure in areas where its use is traditional WHO (1987). The plant shows several biological activities (antimicrobial, dental plaque and gingivitis, anti-inflammatory and analgesic) due to major bioactive compounds like alkaloids, tannins, saponins and sterols (Ahmad et al. 2011; Akhtar et al. 2011; Sofrata et al. 2011a, b). In harsh saline and hot desert conditions, these plants support wild life and are integral part of ecosystem. Details of biology, physiology and usage have been reviewed (Kasera and Mohammed 2010; Khan et al. 2006; Sen et al. 2002).

Cell and tissue culture offers monitoring plant responses to salinity at biochemical and physiological levels (Yang et al. 2010; Matkowaski 2008). In the present study, *S. persica* callus cultures have been used for the first time to investigate salt stress adaptive mechanism, and correlate with antioxidant activity under salt stress for its possible use as a source of antioxidant in salty desert conditions.

## Materials and methods

### Plant material and growth conditions

The explants were obtained from 20- to 30-year-old mature tree of *S. persica* growing near to the University campus. The explants were washed under running tap water for 20 min to remove any adherent particles. Thoroughly washed explants were then immersed in 1 % (v/v) Teepol, a liquid detergent for 2–3 min, and thereafter surface sterilized in ethanol for 30 s. The explants were immersed in 0.1 % (w/v) aqueous solution of HgCl_2_ for 10 min, rinsed with distilled water and then suitable size nodal explants (**~**1 cm) were inoculated on to the medium. Murashige and Skoog (1962) (MS) medium contained 3 % sucrose, 0.8 % agar with different combinations and concentrations of plant growth regulators. The pH was adjusted to 5.8 and autoclaved at 121 °C for 20 min. Organic supplements like coconut milk (10 %) and all vitamins (twofold) were used for the improvement of callus growth and friability. The callus was then separated from the initial explants and subcultured every 30–35 days. The callus was maintained on MS medium with combinations of 2,4,5-T and BAP, 0.5 mg/l each, 3 % (w/v) sucrose, double vitamins and 0.8 % (w/v) agar.

For salt stress experiment, different NaCl concentrations (0, 50, 100 and 200 mM) were added in MS medium and 40 ml medium was poured in 100-ml wide-mouth conical flasks. The callus (2.0 g) was inoculated in each flask and these cultures were incubated at 25 ± 0.2 °C under a 16 h d^−1^ photoperiod with 50 μmol m^−2^ s^−1^ irradiance. Fresh weight (FW) and dry weight (DW) were determined after 30-day growth.

### Soluble protein analysis

The samples were collected from untreated and 30-day NaCl-treated callus for estimation of soluble proteins. The callus (250 mg) was ground in chilled tris-(hydroxymethyl) amino methane (Tris)–HCl buffer (10 mM, pH 6.8), then centrifuged at 15,000*g* for 20 min. The supernatants obtained were used for protein assay according to the method of Bradford (1976) using bovine serum albumin as a standard.

### Proline and soluble carbohydrate assay

The amount of proline was estimated by the ninhydrin method using fresh callus (0.5 g) in 3 % sulfosalicylic acid and organic phase monitored at 520 nm by spectrophotometer (Specord 200, Analyte Jena, Germany) (Bates et al. 1973). A modification of the method of phenol–sulfuric acid was used to determine soluble sugar content at 490 nm (Dubois et al. 1956).

### Catalase activity measurement

Catalase activity was measured by the method of Aebi (1974). The assay system comprised 50 mM phosphate buffer (pH 7.0), 20 mM H_2_O_2_, and a suitable aliquot of enzyme in the final volume of 3 ml. The change of absorption values was recorded at 240 nm for 3 min. CAT activity was estimated by calculating the initial rate of disappearance of H_2_O_2_.

### Determination of antioxidant activity

Antioxidant activity was determined by extraction of samples, which were pooled and analyzed in triplicates. Dried powdered callus sample (200 mg) was extracted 12 h at room temperature by shaking on a test tube rotator with 5 ml of 70 % methanol. The samples were centrifuged at 10,000*g* for 10 min at 10 °C and supernatant was used for antioxidant activities.

### DPPH radical scavenging activity

The 1,1-diphenyl-2-picrylhydrazyl (DPPH) radical scavenging activity was determined according to the method described by Hatano et al. (1988). The reaction mixture (total volume 3 ml), consisting of 0.5 ml of 0.5 M acetic acid buffer solution at pH 5.5, 1 ml of 0.2 mM DPPH in ethanol, and 1.5 ml of 50 % (v/v) ethanol aqueous solution, was shaken vigorously with various samples. After incubation at room temperature for 30 min, the remaining DPPH was determined by absorbance at 517 nm, and the radical scavenging activity of each sample was expressed using the ratio of the absorption decrease of DPPH (%) to that of the control DPPH solution (100 %) in the absence of the sample. The radical scavenging activity was calculated as (%) = 100(*A* − *B*)/*A*, where *A* and *B* are the 517 nm absorption of the control and the corrected absorption of the sample reaction mixture.

### Superoxide radical scavenging activity (PMS/NADH system)

Superoxide anions were generated using PMS/NADH system. The superoxide anions are subsequently made to reduce nitroblue tetrazolium (NBT) which yields a chromogenic product, which is measured at 560 nm. The absorbance was read at 560 nm (Jain et al. 2008) and the inhibition percentage of superoxide anion generation was calculated the same as DPPH activity formula.

### Ferric reducing antioxidant potential (FRAP) assay

The ferric reducing power of plant extracts was determined using the method of Benzie and Strain (1996). The reaction mixture, containing 100 μl of sample solutions, 300 μL of deionized water and 3 ml of FRAP reagent, was incubated for 30 min at 37 °C in a water bath and read at 593 nm. The difference between sample absorbance and blank absorbance was calculated and used to calculate the FRAP value. FRAP values were expressed as mmol Fe^2+^/g of sample.

### Determination of total phenolic content

Phenolic compounds were assayed using the Foline–Ciocalteu reagent, by following the method of Farkas and Kiraly (1962). TPC was expressed as mg gallic acid equivalents (GAE) g^−1^ DW through the calibration curve with gallic acid at 650 nm.

### Statistical analysis

All results were averaged over three separate analyses from six flasks of each treatment and experiment was done in duplicate. Results were reported as the mean ± standard deviation (SD) and analyzed by ANOVA followed by post hoc least significant difference (LSD) test at *P* ≤ 0.05 using prism statistical software. To correlate the results obtained with different methods, a regression analysis was performed and correlation coefficients were calculated.

## Results and discussion

**Fig. 1:**
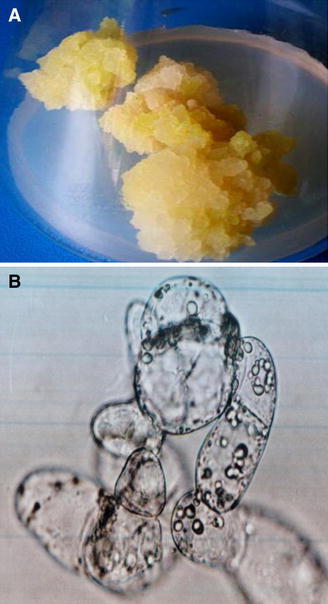
**a** Callus and **b** cells of *Salvadora persica*

The results obtained with callus tissues of *S. persica* grown under NaCl-generated salinity stress showed increased salt tolerance in cells as evident by higher dry weight, metabolic and antioxidant activities. Being a slow growing desert plant, initial growth of the callus produced from mature explants was very slow on different permutations and combinations of MS medium salts, plant growth regulators and complex addendum. However, soft, friable and cream colored cultures could be established after several subcultures on the medium containing a combination of 2,4,5-T and BAP (0.5 mg/l each), 3 % (w/v) sucrose, double vitamins and 0.8 % (w/v) agar. On this medium, metabolically active cells were visible under microscope (Fig. 1a, b). Salt stress creates both ionic as well as osmotic stress on plants.

### Effect of NaCl on growth and soluble protein contents

Reduced fresh weight with higher or same dry weight and protein contents were observed in the callus after 30-day growth on the medium with NaCl (Table 1). Loss of water with high salt concentration but increased dry matter and soluble protein showed high metabolic activity.

**Table 1:** Effect of NaCl on the growth and soluble proteins of callus culture of *Salvadora persica*

Growth	NaCl concentrations (mM)^*#^			
	Control	50	100	200
Relative fresh weight (%)	100 ± 1.20^a^	91.66 ± 1.05^b^	83.33 ± 1.15^c^	66.66 ± 1.54^d^
Dry weight (mg)	510 ± 18.0^bc^	580 ± 15.5^a^	540 ± 20.3^b^	500 ± 12.6^c^
Soluble protein (mg g^−1^ FW)	1.0 ± 0.06^c^	1.9 ± 0.10^a^	1.8 ± 0.07^a^	1.6 ± 0.13^b^

* Values represented mean ± SD calculated from at least three replicates of each treatment

^#^Mean with common letter is not significantly different at *P* ≤ 0.05, according to least significant difference (LSD) test

Fresh weight and dry weight are often measured to reveal the growth of plants and cells in response to environmental stresses. In the present study, salt concentrations induced significant elevation in dry weight in *S. persica* callus, which suggest a cellular tolerance to lower salinity in this halophytic species. Similar results of growth were found in *Nitraria tangutorum* and *Oryza sativa* callus (Yang et al. 2010). The expression of proteins is regulated in plants depending on salt concentration (Sekmen et al. 2007; Yang et al. 2010). Proteins that accumulate in plants under saline conditions are cytoplasmic, which can cause alterations in cytoplasmic viscosity of the cells and may play a significant role in osmotic adjustment (Hasegawa et al. 2000). Similar results were also obtained in mulberry cultivars where soluble protein increased at low salinity and decreased at high salinity (Agastian et al. 2000).

### Effect of NaCl stress on proline and soluble sugar contents

**Fig. 2:**
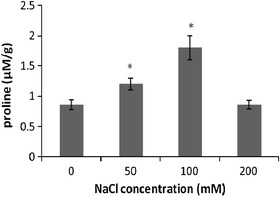
The change of proline contents induced by NaCl treatment in the callus of *S. persica*. Data represent the mean ± SE (*n* = 3). *Significant differences between the control and treated callus (*P* < 0.05)

**Fig. 3:**
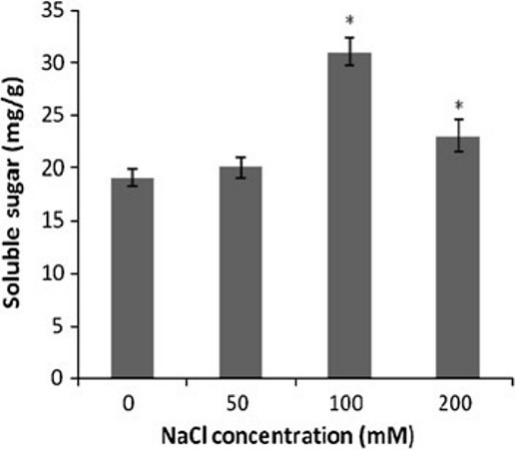
The change of soluble sugar contents induced by NaCl treatment in the callus of *Salvadora persica*. Data represent the means ± SE of at least three independent measurements. *Significant differences between the control and treated callus (*P* < 0.05)

Proline level in response to NaCl treatment (Fig. 2) showed an approximately 1.30- and 2.09-fold increase in the content in the callus exposed to 50 and 100 mM NaCl, respectively (*P* < 0.05), as compared with control cultures. However, treatment with 200 mM NaCl led to a decrease in proline content which was equivalent to that of control value. In callus cultures of *S. persica*, soluble sugar content increased significantly from 1.0- to 1.6-fold under salinity stress (*P* < 0.05; Fig. 3). Therefore, it appears that proline possibly plays a more important role as an osmoprotectant in *S. persica* callus subjected to salinity stress than to soluble sugar.

Proline is known to play an important role as an osmoprotectant and is accumulated when plants are subjected to hyperosmotic stresses (Suriyan and Chalermpol 2009). Maintaining osmotic homeostasis by accumulating metabolically compatible compounds such as carbohydrates and proline may be among the important adaptive mechanisms of salinity tolerance in plants (Rosa et al. 2009). Increased proline and sugar contents were correlative with increased salinity stress in the callus of *S. persica*. In *Sesuvium portulacastrum*, treatment with 100 mM NaCl induced significantly increased levels of proline in the leaves, but it had no effect on soluble sugar content (Slama et al. 2007). Proline is also considered to be involved in the protection of enzymes, cellular structures, and to act as a free radical scavenger (Aghaleh et al. 2011).

### Effect of NaCl on CAT activity

**Fig. 4:**
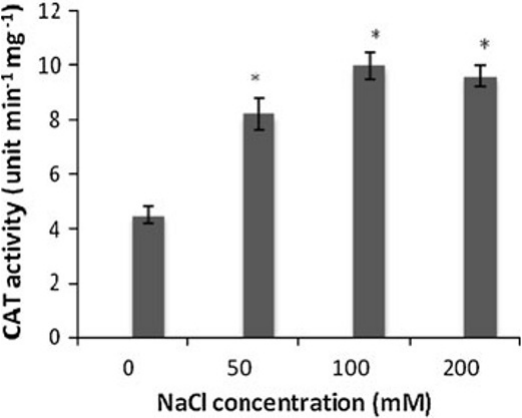
The changes in CAT activity in *S. persica* callus treated with different NaCl concentrations. Data represent the mean ± SE (*n* = 3). *Significant differences between the control and NaCl treated callus (*P* < 0.05)

An increase in 82, 122 and 113 % CAT activity was measured in the callus grown on 50, 100 and 200 mM NaCl containing medium for 30 days, respectively, (*P* < 0.05), as compared to control culture values (Fig. 4).

An effective ROS-scavenging system involving catalase is a critical component of salinity resistance because of its protective effect against oxidative damage under salinity stress. Catalase is one of the main H_2_O_2_ scavenging enzymes that dismutate H_2_O_2_ into water and O_2_ (Moradi and Ismail 2007; Li 2008). When *Bruguiera parviflora* plants were subjected to 400 mM NaCl stress condition, a decrease in total catalase activity was observed by Jitesh et al. (2006), while increased CAT activity was demonstrated in *Nitraria tangutorum* callus culture after 50 or 100 mM NaCl treatment (Yang et al. 2010). Thus, catalase enzyme activity varied with different plant species and with salt stress.

### Effect of NaCl stress on antioxidant potential and total phenolic content

Antioxidant capacity was evaluated by DPPH and SOD anion activities. DPPH radical scavenging activity of callus cultures of *S. persica* increased gradually from 1.26- to 1.78-fold when grown on increasing concentrations of NaCl for 30 days (Table 2). FRAP and SOD activities of *S. persica* callus cultures were also increased steadily from 9 to 12 % and 11 to 17 mM Fe^2+^ g^−1^ under 50 to 200 mM NaCl treatments, respectively (Table 2). Increased protein and metabolites were correlative to increased TPC, DPPH, SOD and FRAP in the cells grown with increased salt stress as compared to control. The plant cells showed increase in all parameters under increased stress indicating a salt-induced response for survival in harsh temperature and salt conditions with increase of dry matter accumulation. Simultaneously, TPC of the callus cultures under salinity stress increased from 11 to 12 mg GAE g^−1^ in the cultures grown on the medium with increased concentration of NaCl as compared to control cultures (Table 2).

**Table 2:** Effect of NaCl on the total phenolic content (TPC) and radical scavenging capacity of callus culture of *Salvadora persica*

TPC and antioxidant activity	NaCl concentrations (mM)*#			
	Control	50	100	200
TPC (mg GAE g^−1^)	10 ± 0.45^b^	11 ± 0.70^a^	11 ± 0.34^a^	12 ± 0.86^a^
DPPH scavenging activity (%)	46 ± 2.3^d^	58 ± 3.0^c^	65 ± 3.4^b^	82 ± 4.1^a^
SOD quenching (%)	6 ± 0.37^c^	9 ± 0.45^b^	10 ± 0.40^b^	12 ± 0.92^a^
FRAP activity (mM Fe^2+^ g^−1^)	8 ± 0.66^d^	11 ± 0.40^c^	13 ± 0.55^b^	17 ± 1.03^a^

* Values represented mean ± SD calculated from at least three replicates of each treatment

^#^Mean with common letter is not significantly different at *P* ≤ 0.05, according to least significant difference (LSD) test

### Correlation between assays

**Fig. 5:**
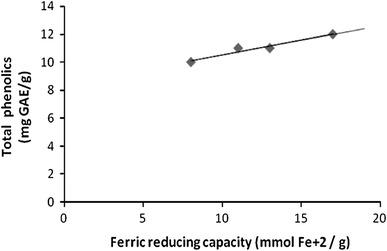
Correlation between ferric reducing capacity (FRAP) and total phenolic content (TPC). Correlation coefficient *R* = 0.973

**Fig. 6:**
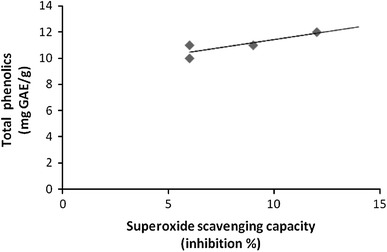
Correlation between superoxide scavenging capacity (SOD) and total phenolic content (TPC). Correlation coefficient *R* = 0.852

**Fig. 7:**
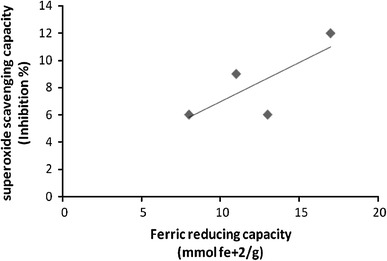
Correlation between superoxide scavenging capacity (SOD) and ferric reducing capacity (FRAP). Correlation coefficient *R* = 0.760

In order to correlate the results obtained with the different methods, a regression analysis was performed (correlation coefficient *R*, Table 3). Significant correlations were established between FRAP and TPC (*R* = 0.973, Fig. 5), and SOD and TPC (*R* = 0.852, Fig. 6) assay. A significant correlation (*R* = 0.760, Fig. 7) was also observed between FRAP and SOD assay. However, the negative correlations were found between FRAP and DPPH (*R* = −0.164), SOD and DPPH (*R* = −0.532) and TPC and DPPH (*R* = −0.153) assays. The negative correlation coefficient of above assays shows that as the value of one variable increases, the value of other variable decreases, and vice versa. The DPPH activity commonly showed negative correlation with FRAP, SOD and TPC.

**Table 3:** Correlation coefficient (*R*) between assays

	FRAP	SOD	DPPH
SOD	0.760		
DPPH	−0.164	−0.532	
TPC	0.973	0.852	−0.153

Salt-induced growth variations were correlated with parallel variations in polyphenols accumulation and antioxidative ability. NaCl-treated callus of *S. persica* demonstrated that DPPH radical scavenging activity, FRAP activity and SOD activity increased significantly. Similarly, Lechno et al. (1997) reported that NaCl treatment increases the activities of the antioxidative enzymes. These activities may be directly linked to the content of phenols, tannins and flavonoids and consequently to their free radical scavenging activities. Besides, plant resistance to various stresses is associated with antioxidant capacity and increased levels of antioxidants may prevent stress damage (Bettaieb et al. 2011).

These results indicate a significant correlation between TPC in callus extract and their free radical scavenging and ferric reducing capacities (FRAP and SOD assays). Therefore, the presence of phenolic compounds in callus extracts contributed significantly to their antioxidant potential. In our experiments, results demonstrated a strong correlation between TPC and FRAP activity. Similarly, previous studies suggested that ferric reducing capacity can be related to phenolic content, indicating that phenolic compounds are the major contributor to the antioxidant potential of different plant extracts (Dudonne et al. 2009).

In conclusion, our results showed that salinity-induced in vitro cultures are better model system for the study of stress mechanism, which is independent from environmental factors. The callus cultures of *S. persica* showed moderate salt-tolerant properties and can be used as source of antioxidants in harsh saline desert conditions for humans (fruits) and cattle (leaves).

## Abbreviations

BAP: 6-Benzyl aminopurine
ROS: Reactive oxygen species
SOD: Superoxide dismutase
CAT: Catalase
2,4,5-T: 2,4,5-Trichlorophenoxy acetic acid
H_2_O_2_: Hydrogen peroxide
DPPH: 1,1-Diphenyl-2-picrylhydrazyl
FRAP: Ferric reducing antioxidant potential
TPC: Total phenolic content
